# VV-ECMO combined with prone position ventilation in the treatment of Pneumocystis jirovecii pneumonia: A case report

**DOI:** 10.1097/MD.0000000000028482

**Published:** 2022-01-07

**Authors:** Lijing Jia, Zhiyang Zhang, Yinxiang Bai, Quansheng Du

**Affiliations:** Intensive Care Unit, Hebei General Hospital, Shijiazhuang City, Hebei Province, PR China.

**Keywords:** Pneumocystis jirovecii pneumonia, prone position ventilation, veno-venous extracorporeal membrane oxygenation

## Abstract

**Introduction:**

Pneumocystis jirovecii pneumonia (PJP) occurs in immunocompromised hosts. It is classified as PJP with human immunodeficiency virus (HIV) infection (HIV-PJP) and PJP without HIV infection (non-HIV PJP). Compared with HIV-PJP, non-HIV PJP is more likely to develop rapidly into respiratory failure, with difficult diagnosis and high mortality.

**Patient concerns:**

A 46-year-old male with membranous nephropathy was treated with oral corticosteroids and tacrolimus. He was admitted to our hospital for fever and dyspnea which developed 4 days ago. Laboratory data revealed that leukocytes were 10.99 × 10^9^/L, neutrophils 87.7%, lymphocytes 9.6%, C-reactive protein 252.92 mg/L, New coronavirus nucleic acid detection negative. CT scan of chest revealed ground-glass opacity in both lungs. He was admitted to the respiratory department of our hospital, and then transferred to ICU because of his critical condition.

**Diagnosis:**

High throughput gene detection of pathogenic microorganisms in alveolar lavage fluid showed that the detection sequence of Pneumocystis yersiniae increased significantly. The serum HIV-antibody was negative. Therefore, the patient was diagnosed as non-HIV PJP.

**Interventions:**

After admission, the patient was assisted by noninvasive ventilator and treated with compound trimethoprim-sulfamethoxazole (SMX-TMP) and caspofungin. The patient's condition continued to deteriorate, and then underwent endotracheal intubation and veno-venous extracorporeal membrane oxygenation (VV-ECMO) combined with prone position ventilation until the lung lesion improved.

**Outcomes:**

VV-ECMO was stopped on day 12, tracheal intubation was removed after 2 days. The patient was transferred to the respiratory department on day 15, discharged after 12 days without complications. Two months later, the follow-up showed that the patient was in good condition.

**Conclusion:**

VV-ECMO combined with prone position ventilation could be a useful choice for respiratory assistance in non-HIV PJP patients.

## 1 Introduction

Pneumocystis jirovecii pneumonia (PJP) is an infectious respiratory disease that occurs in immunocompromised hosts. PJP is clinically classified as PJP with human immunodeficiency virus (HIV) infection (HIV-PJP) and PJP without HIV infection (non-HIV PJP) because of the difference in therapeutic strategy and prognosis. Non-HIV PJP could occur in patients with immunodeficiency status without HIV infection, such as post-organ transplants, malignant diseases, and chronic inflammatory diseases under immunosuppressant therapy. The prognosis of PJP in non-HIV infected patients is usually worse than that of PJP in HIV infected patients.^[1]^ PJP in non-HIV infected patients often results in fatal respiratory failure.^[2]^The number of PJP cases in non-HIV-infected patients is increasing in association with the increase in the number of patients undergoing chemotherapy, organ transplantation, and autoimmune diseases.^[3–7]^ The overall mortality for patients with non-HIV PJP is approximately 30%.^[2]^ Moreover, overall mortality of patients with non-HIV PJP who are admitted to the intensive care unit (ICU) is reported to be 75.6%.^[8]^

Extracorporeal gas exchange is a technique that enables gas exchange by the extracorporeal circulation of blood through an artificial organ.^[9]^ Veno-venous extracorporeal membrane oxygenation (VV-ECMO) is a mechanical-assisted therapy for the extracorporeal gas exchange in patients with severe respiratory failure, which oxygenates and removes carbon dioxide from the blood.^[10,11]^ As non-HIV PJP more frequently progresses to respiratory failure compared with HIV-PJP, non-HIV PJP could be the indication for VV-ECMO assistance. Little is known, however, about the efficacy of VV-ECMO for cases of non-HIV PJP. It is important to know the clinical course and prognosis of non-HIV PJP who are managed by VV-ECMO. PPV is a common treatment for acute respiratory distress syndrome (ARDS) patients. and can also significantly improve PaO_2_ and oxygenation index in patients with severe pneumonia, and significantly reduce PaCO_2_.^[12]^

Herein, we report a case of non-HIV PJP who were successfully managed with VV-ECMO combined with prone position ventilation (PPV).

## 2 Case presentation

Written informed consent was obtained from the patient for publication of this case report and any accompanying images. A copy of the written consent is available for review by the Editor-in-Chief of this journal. Because of this, there is no need to conduct special ethic review and the ethical approval is not necessary.

**Figure 1:**
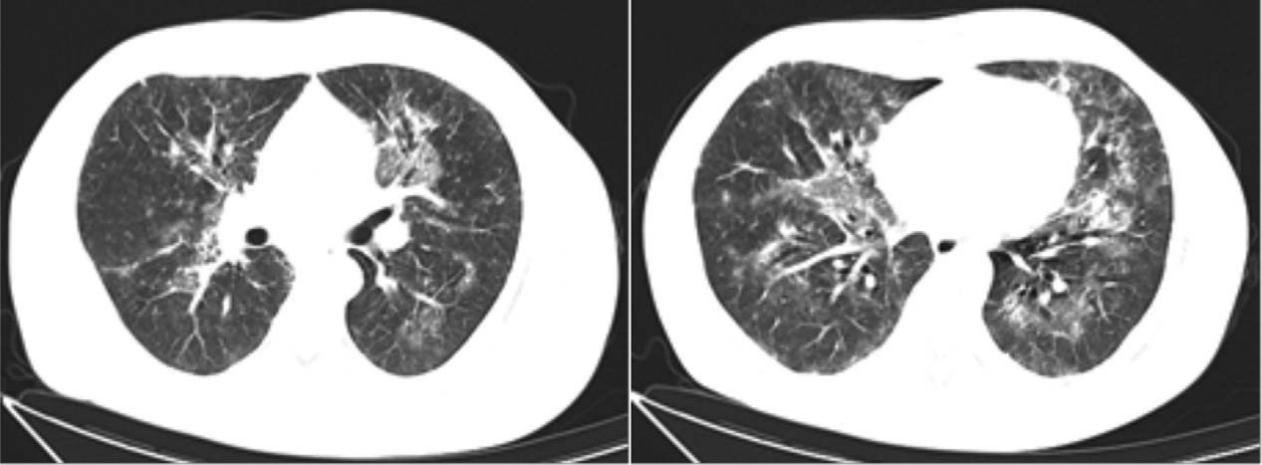
CT scan of the chest in outpatient department. CT scan showed exudative lesions in both lungs.

A 46-year-old man, went to the outpatient department of our hospital complaints of fever (the highest body temperature is 39.7°C) and shortness of breath 4 days after activity. Laboratory data revealed that leukocytes were 10.99 × 10^9^/L, neutrophils 87.7%, lymphocytes 9.6%, C-reactive protein 252.92 mg/L, New coronavirus nucleic acid detection negative. CT scan of the chest showed double lung inflammation (Fig. 1). He was admitted to the respiratory department of our hospital on July 26, 2021 with “lung infection.” The patient had a history of membranous nephropathy for 1 year and was treated with ethylprednisolone tablets and tacrolimus capsules in recent 2 months. There was no history of genetic disease or infectious disease in the family. After admission, the patient's body temperature was 39.1°C, his mind was clear, his lips were cyanotic, double lung auscultation breathing sounds were thick, wet rales could be heard. After admission, blood gas analysis showed that the patient had obvious hypoxia (nasal catheter oxygen inhalation 5 L/min, pH7.41, PaO_2_ 49.40 mm Hg, PaCO_2_ 25.90 mm Hg, HCO_3_^-^16.20 mmol/L, BE-6.88 mmol/L, Lac3.80 mmol/L, SaO_2_ 84.40%), and high procalcitonin (1.87 ng/mL) and interleukin-6 (160.0 pg/mL).

**Figure 2:**
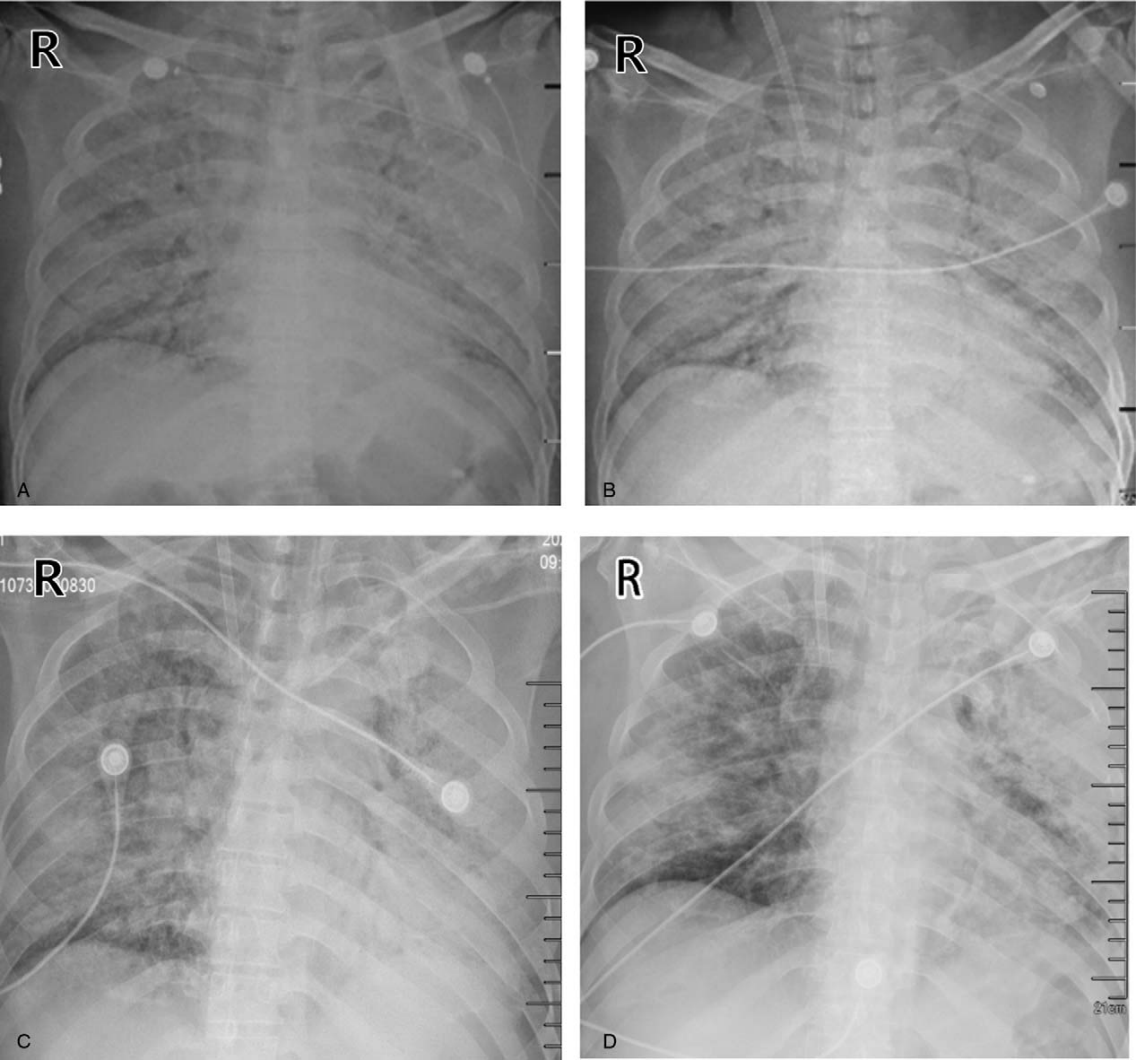
Bedside chest X-ray on the day 2,3,5, and 8. (A) Chest X-ray showed that the exudative lesions of both lungs were more severe than before on day 2. (B) Chest X-ray was rechecked after ECMO catheterization on day 3. (C) and (D) Chest X-ray showed that the lesions of the right lung were lighter than before, and the left were slightly lighter.

The patient had severe pneumonia and was assisted by noninvasive ventilator after admission (mode S/T, IPAP 10cmH_2_O, EPAP 4cmH_2_O, FiO_2_80%). Considering that the etiology of the patient was not clear, etiological examination was actively carried out after admission. Combined with the patient's history of membranous nephropathy, long-term oral hormone, and immunosuppressant treatment, he was considered to be the immunosuppressive host. The pathogens may be gram-positive cocci, gram-negative bacilli, mycoplasma pneumoniae, viruses, fungi (including Pneumocystis carinii). After admission, the patient was treated with meropenem, moxifloxacin, oseltamivir phosphate, trimethoprim-sulfamethoxazole, and caspofungin. Nucleic acid detection of influenza A and B viruses, nucleic acid detection of cytomegalovirus and EB virus, acid fast staining, 1-3-β-D glucan and aspergillus immune test results showed no abnormality, so oseltamivir phosphate was stopped on day 2. On the same day, the patient's dyspnea worsened (the respiratory rate 40–50 breaths min^–1^), the oxygen concentration of noninvasive ventilator was increased to 100%, but the symptoms were not relieved. Bedside chest X-ray showed that the exudation of both lungs was obvious (Fig. 2A). So, the patient received endotracheal intubation and invasive ventilator to assist breathing. The patient's oxygenation index continued to decline, lower than 80 mm Hg. The patient's condition was very critical. According to ARDS criteria,^[13]^ the patient was considered to have severe ARDS. The ECMO team of the ICU performed VV-ECMO support treatment for the patient on day 3 (review bedside chest X-ray after catheterization, Fig. 2B), and then transferred to ICU under the protection of ECMO.

After being transferred to ICU, we evaluated the patient's condition. Acute Physiology and Chronic Health Evaluation II was 22, Sequential Organ Failure Assessment was 10, Critical Nutritional Risk (NUTRIC score) was 4, and Venous Thromboembolism Risk (Padua score) was 6. In the early stage of ECMO support, volume controlled ventilation (Vt300 mL, PEEP10cmH_2_0, F10breaths min^–1^, FiO_2_50%) was used under deep sedation, that is, critical care pain observation tool 0 point and Ramsay sedation scores –4 point. However, the oxygen saturation of the patient was still below 90%, so the PPV was adopted, and the prone position time was 16 to 20 hours a day. On day 5 (the 3rd day of ICU treatment), high-throughput gene detection of pathogenic microorganisms reported that the number of detected sequences of Pneumocystis jirovecii and Acinetobacter baumannii increased significantly, followed by Elizabeth meningitidis and Serratia marcescens. Mycobacterium tuberculosis, mycoplasma, Chlamydia, and virus sequences were not detected. The serum HIV-antibody was negative. So the patient was diagnosed as non-HIV PJP. Moxifloxacin was stopped, SMX-TMP (3 tablets q6 hour) combined with caspofungin (50 mg once a day) were used to treat Pneumocystis jirovecii, and meropenem (1 g q8 hour) was used to treat bacterial infection. After that, the results of alveolar lavage fluid culture were reported intermittently, including Pseudomonas aeruginosa (resistant to meropenem) and Stenotrophomonas maltophilia (sensitive to SMX-TMP) on August 1, Klebsiella pneumoniae (resistant to meropenem) and Pseudomonas aeruginosa (sensitive to meropenem) on August 4, and Stenotrophomonas maltophilia (resistant to SMX-TMP) and Pseudomonas aeruginosa (sensitive to meropenem) on August 6. Although some bacteria cultured in lung lavage fluid were resistant to the antibiotics used, the patient's body temperature and oxygenation were improved, so the antibiotics were not adjusted. Lymphocyte immunoassay: total T lymphocyte count (CD3+) 490/UL (normal value 470-3260/UL), auxiliary/induced T cell count (CD3+CD4+) 342/UL (normal value 350-1820/UL), inhibitory/cytotoxic T cell count (CD3 + CD8+) 127/UL (normal value 130-1350/UL), NK cell count (CD3-CD16 + CD56) 180/UL (normal value 40-1000/UL), B cell count 23/UL (normal value 50-670/UL). The counts of T lymphocytes and B cells were low, suggesting that there was low immune function. Considering that the patient had membranous nephropathy, obvious exudation of both lungs and poor immune function, dexamethasone (20 mg once a day for 5 days, then 10 mg once a day for 5 days, and then gradually reduced to the previous oral dose of 16 mg once a day) and immunoglobulin (20 g once a day for 3 days) were added. The patient had a low nutritional risk, but he had a higher risk of stomach retention and reflux because of PPV, so early enteral nutrition support was given by nasal intestinal tube. The patient had a high risk of venous thromboembolism. During the anticoagulation period of ECMO, bilateral lower extremity pneumatic pump treatment were given to prevent thrombosis, and low molecular weight heparin was added after ECMO was stopped.

**Figure 3:**
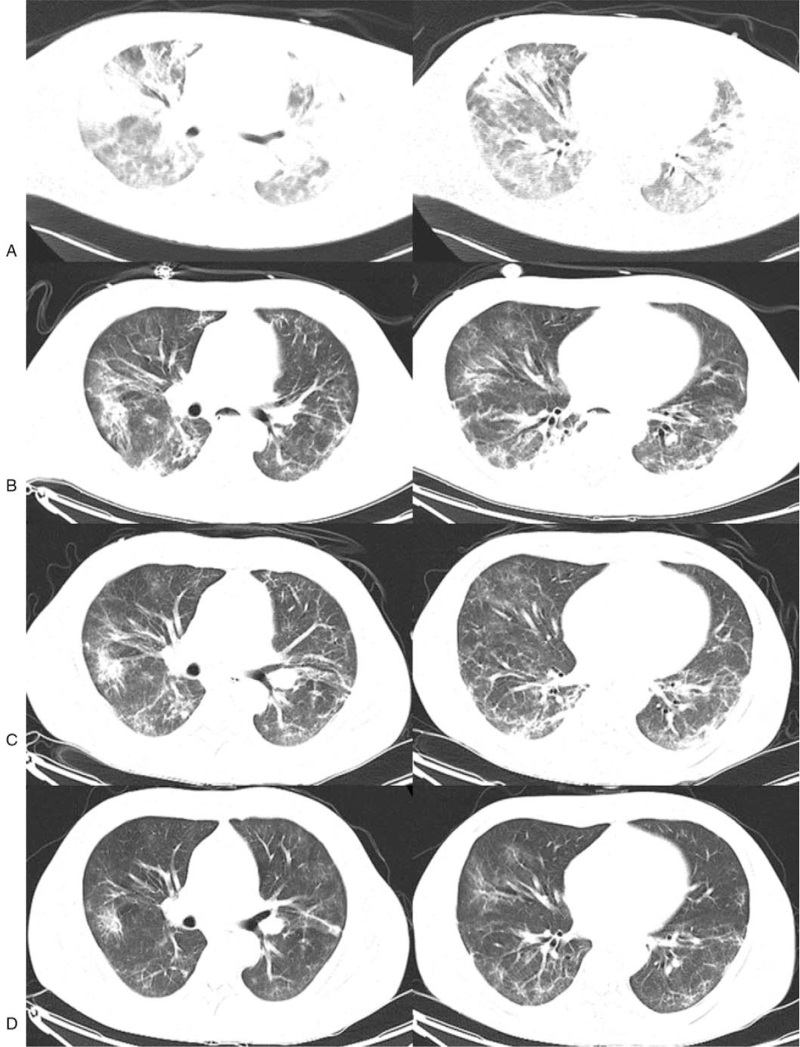
CT scans of the chest on day 9, 13, 17, and 24. (A)-(D) CT scans of the chest showed that lung lesions gradually improved.

After treatment, the patient's oxygenation improved, the peak airway pressure decreased, and the pulmonary static compliance increased (Table 1). Leukocytes, high procalcitonin, C-reactive protein, and interleukin-6decreased to normal. On day 10, the patient stopped prone ventilation, adjusted the depth of sedation to shallow sedation (CPOT 0, RASS –2 to 0), and adjusted the ventilator mode to synchronized intermittent mandatory ventilation (SIMV PC 10cmH_2_0, PS 10cmH_2_0, PEEP 8cmH_2_0, F12 breaths min^–1^, FiO_2_40%). The changes of ECMO and ventilator parameters are shown in Table 1. ECMO support was stopped on day 12, tracheal intubation was removed after 2 days, and sequential transnasal high flow oxygen inhalation was applied. The patient was transferred to the respiratory department on day 15 and discharged after 12 days. During the treatment, we intermittently used bedside chest X-ray and CT scans of chest to understand the pulmonary lesions. Bedside chest X-ray showed that the right lung lesions are less severe than before on day 5 and 8 (Fig. 2C, 2D). After the patient's condition was slightly stable, we began to perform CT scans of chest intermittently, and the results showed that the lung lesions gradually improved (Fig. 3). During the treatment, the patient's tolerance and compliance were good, and no adverse events occurred. Two months later, follow-up showed that the patient was in good condition.

**Table 1:** Changes of ECMO parameters, ventilator parameters, blood gas analysis, P_peak_, and Cst.

Project	Before ECMO	D1	D3	D5	D7	D9	D11
ECMO parameters							
Pump speed(rpm)	–	2800	3000	2800	2200	2300	–
Flow(L/min)	–	4.5	4.5	4.5	3.2	3.6	–
Sweep(L/min)	–	5	4	6	6	4.5	–
FiO_2_	–	100	100	80	60	30	–
Ventilator parameters							
Ventilator mode	PSV	VCV	VCV	VCV	SIMV(PC) + PSV	SIMV(PC) + PSV	PSV
VT(mL)	–	300	300	300	–	–	–
RR(per min)	–	10	10	10	12	12	–
FiO_2_(%)	100	50	40	40	40	40	40
PEEP(cmH_2_O)	9	10	10	10	8	8	6
Blood gas analysis							
PaO_2_(mm Hg)	70.5	73.5	76.9	75.5	75.3	112.5	135.5
PaCO_2_(mm Hg)	41.6	38.2	43.6	40.9	36.1	43.2	39
Lac(mmol/L)	2.3	1.8	1.5	1.6	1.4	1.1	<1.0
P_peak_(cmH_2_0)	–	30	24	21	21	18	14
Cst(mL/cmH_2_0)	–	20	20	47	63	–	–

## 3 Discussion

This is a case of severe ARDS caused by non-HIV PJP. In clinical practice, it is very difficult to diagnose and treat the disease.

The patient has a history of membranous nephropathy, usually takes oral hormones, immunosuppressants and other drugs, and has been hospitalized repeatedly in recent years. This time, the patient was hospitalized in the community. In addition to the common pathogens of community-acquired pneumonia, the patient is easy to be complicated with pathogens that are easy to cause hospital-acquired infection, such as Klebsiella pneumoniae, Pseudomonas aeruginosa, and Acinetobacter baumannii. In addition, there may also be opportunistic pathogen infection, such as Mycobacterium tuberculosis infection, Yersinia pneumocystis infection, etc. Therefore, etiological diagnosis has become the primary problem faced by clinicians. In terms of etiological diagnosis, we early carried out high-throughput gene detection of pathogenic microorganisms in alveolar lavage fluid to assist in diagnosis, so as to clarify etiology as soon as possible and provide basis for treatment.

TMP-SMZ have been used as the first-line drug against PJP for a long time, but PJP resistant to TMP-SMZ has increased in recent years.^[14]^ Caspofungin can inhibit the growth of fungi on the cell wall β-(1,3)-D-glucan synthesis leads to the destruction of the integrity and permeability of fungal cell wall and cell lysis. The cell wall of Yersinia (PJ) also contains β-(1,3)-D-glucan, so caspofungin is also effective for PJ. TMP-SMZ interferes with the nutrition of PJ. Caspofungin is effective for the capsule. TMP-SMZ combined with caspofungin can inhibit the whole life cycle of PJ, which can play a synergistic effect against PJ.^[15]^

Mechanical ventilation is the basic treatment for patients with severe ARDS, but the traditional mechanical ventilation mode is prone to secondary lung injury due to uneven gas distribution in the lung and excessive expansion of some alveoli. In recent years, ECMO has become an important treatment for patients with severe ARDS. According to different ways of blood reinfusion, ECMO usually has 2 types: VA-ECMO led from venous system and arterial reinfusion; VV-ECMO was introduced from the vein and then injected into the vein. VV-ECMO is required for patients with severe ARDS and hypoxia that cannot be corrected by mechanical ventilation. VV-ECMO can improve gas exchange, quickly correct hypoxemia and hypercapnia, and provide a time window for the treatment of primary diseases; reduce the huge changes of trans pulmonary pressure caused by strong spontaneous breathing and its related lung injury. Under the support of VV-ECMO, it is allowed to reduce the strength of mechanical ventilation support, so as to reduce ventilator-related lung injury.^[16]^

For patients with severe ARDS, during VV-ECMO support, due to the lung protective ventilation strategy of low tidal volume, some atelectasis will be caused.^[17]^ Therefore, despite the application of VV-ECMO auxiliary support, it is still insufficient to maintain its oxygenation demand, and oxygenation may still be low.^[9]^ In conclusion, we implemented PPV for patients during VV-ECMO. PPV can increase the recruitment ventilation of the previously collapsed atelectasis dorsal lung and improve the ventilation/blood flow ratio of the overall lung tissue.^[18]^ It is also conducive to the drainage of pulmonary secretions, especially in the lower lobes and dorsal lungs, so as to improve ventilation.^[19]^ On the basis of VV-ECMO combined with PPV, the patient significantly improved the oxygenation status and pulmonary static compliance, and there were no adverse events. However, in the actual operation process, the maintenance of ECMO pipeline shall be fully considered to prevent complications such as disconnection, discount and bleeding at the place of pipe placement.

The patient said that if he could realize that the disease would progress to such a serious extent, he would go to the hospital earlier. It can be seen that in our daily work, we should strengthen the health education for such patients with low immune function and improve their attention to the disease.

## 4 Conclusion

There are few reports on the application of VV-ECMO combined with PPV in the treatment of severe ARDS caused by PJP. For critical patients with unclear etiology, we applied high-throughput gene detection of pathogenic microorganisms in the early stage to identify pathogenic microorganisms as soon as possible, and actively applied VV-ECMO combined PPV to support treatment, which achieved good therapeutic effect and no adverse events. This suggests that the treatment of VV-ECMO combined with PPV is effective and safe. However, the deficiency is that due to the limited number of cases of ARDS treated with ECMO, the indication, treatment duration, and termination criteria of ECMO combined with PPV are not clear. The effect of this treatment method needs to be further evaluated in later research.

## Author contributions

**Project administration:** Sheng Quan Du.

**Writing – original draft:** Jing Li Jia.

**Writing – review & editing:** Jing Li Jia, Yang Zhi Zhang, Xiang Yin Bai.
